# Supplementary material for: Antibiotic Use, Healthcare-Associated Infections, and Antimicrobial Resistance in Intensive Care Unit of a Serbian Tertiary University Hospital, 2018–2024: An Ecological Analysis
Source: Antibiotics (Basel). 2025 Nov 4;14(11):1110. doi: 10.3390/antibiotics14111110 (PMC12649740; doi:10.3390/antibiotics14111110)
Supplement: Supplementary file 1 [file antibiotics-14-01110-s001.zip › antibiotics-3924133-supplementary.pdf]

Supplementary Table S1. ICD-10 diagnostic categories of ICU patients included in the study

	Year								
Characteristics; N (%)	2018 N=238	2019 N=236	2020 N=298	2021 N=298	2022 N=279	2023 N=367	2024 N=339	Total N=2055	p
**Infectious and parasitic diseases**	3	0	1	2	0	4	1	11	/
	1.3	0.0	0.3	0.7	0.0	1.1	0.3	0.5	
**Neoplasms**	27	48	43	43	48	53	52	314	0.540
	11.3	20.3	14.4	14.4	17.2	14.4	15.3	15.3	
**Diseases of the blood and**	5	14	5	6	10	9	8	57	1.000
**blood-forming organs**	2.1	5.9	1.7	2.0	3.6	2.5	2.4	2.8	
**Diseases circulatory system**	69	49	78	44	67	93	95	495	0.704
	29.0	20.8	26.2	14.8	24.0	18.8	28.0	24.1	
**Endocrine, nutritional and**	2	0	1	2	0	2	0	7	/
**metabolic diseases**	0.8	0.0	0.3	0.7	0.0	0.5	0.0	0.3	
**Diseases of the respiratory**	2	0	5	7	7	5	5	31	0.134
**system**	0.8	0.0	1.7	2.3	2.5	1.4	1.5	1.5	
**Diseases of the nervous system**	3	2	5	0	3	2	5	20	/
	1.3	0.8	1.7	0.0	1.1	0.5	1.5	1.0	
**Diseases of the digestive system**	62	47	86	83	69	84	77	505	0.254
	26.1	19.1	28.5	27.9	24.7	22.9	22.7	24.6	
**Diseases of the skin and**	2	4	5	6	4	9	4	34	0.561
**subcutaneous tissue**	0.8	1.7	1.7	2.0	1.4	2.5	1.2	1.7	
**Diseases of the musculoskeletal**	1	6	4	3	3	3	5	25	1.000
**system and connective tissue**	0.4	2.5	1.3	1.0	1.1	0.8	1.5	1.2	
**Diseases of the genitourinary**	3	4	3	8	5	3	5	31	1.000
**system**	1.3	1.7	1.0	2.7	1.8	0.8	1.5	1.5	
**Congenital malformation**	0	0	0	2	1	0	2	5	/
	0.0	0.0	0.0	0.7	0.4	0.0	0.6	0.2	
**Injury**	42	49	54	54	50	78	67	394	0.333
	17.6	20.8	18.1	18.1	17.9	21.3	19.8	19.2	
**Poisoning**	15	8	6	19	10	15	11	84	0.704
	6.3	3.4	2.0	6.4	3.6	4.1	3.2	4.1	
**External cause and Factors**	2	5	2	18	2	7	2	38	/
**influence health status and**	0.8	2.1	0.7	6.0	0.7	1.9	0.6	1.8	
**contact with health services**									

ICD-10 — International Statistical Classification of Diseases and Related Health Problems, 10th Revision. This table lists the primary diagnostic categories of patients admitted to the surgical intensive care unit (ICU) during the study period. Each entry corresponds to the ICD-10 code assigned at admission, grouped by major clinical category (surgical, neurological, cardiovascular, or infectious). These details provide context for interpreting infection risk and antibiotic consumption patterns reported in the main manuscript.
